# Target and drug predictions for SARS-CoV-2 infection in hepatocellular carcinoma patients

**DOI:** 10.1371/journal.pone.0269249

**Published:** 2022-05-31

**Authors:** Luhong Wang, Yinan Ding, Chuanyong Zhang, Rong Chen

**Affiliations:** 1 Southeast University Medical College, Nanjing, Jiangsu, China; 2 Department of Oncology, Zhongda Hospital, Southeast University, Nanjing, Jiangsu, China; 3 Key Laboratory of Living Donor Liver Transplantation of Ministry of Public Health, Department of Liver Transplantation, First Affiliated Hospital of Nanjing Medical University, Nanjing, Jiangsu Province, China; Hamad Medical Corporation, QATAR

## Abstract

Severe acute respiratory syndrome coronavirus 2 (SARS-CoV-2) is the cause of the coronavirus disease (COVID-19), which poses a major threat to humans worldwide. With the continuous progress of the pandemic, a growing number of people are infected with SARS-CoV-2, including hepatocellular carcinoma (HCC) patients. However, the relationship between COVID-19 and HCC has not been fully elucidated. In order to provide better treatment for HCC patients infected with SARS-CoV-2, it’s urgently needed to identify common targets and find effective drugs for both. In our study, transcriptomic analysis was performed on both selected lung epithelial cell datasets of COVID-19 patients and the datasets of HCC patients to identify the synergistic effect of COVID-19 in HCC patients. What’s more, common differentially expressed genes were identified, and a protein-protein interactions network was designed. Then, hub genes and basic modules were detected based on the protein-protein interactions network. Next, functional analysis was performed using gene ontology terminology and the Kyoto Encyclopedia of Genes and Genomes pathway. Finally, protein-protein interactions revealed COVID-19 interaction with key proteins associated with HCC and further identified transcription factor (TF) genes and microRNAs (miRNA) with differentially expressed gene interactions and transcription factor activity. This study reveals that COVID-19 and HCC are closely linked at the molecular level and proposes drugs that may play an important role in HCC patients with COVID-19. More importantly, according to the results of our research, two critical drugs, Ilomastat and Palmatine, may be effective for HCC patients with COVID-19, which provides clinicians with a novel therapeutic idea when facing possible complications in HCC patients with COVID-19.

## Introduction

Currently, the world is experiencing a serious pandemic due to the outbreak of coronavirus disease (COVID-19). The pandemic was caused by severe acute respiratory syndrome coronavirus 2 (SARS-CoV-2) [1]. As far as we know, Angiotensin-converting enzyme 2, the functional receptor of SARS-CoV-2, is the crux of the viral infection [2]. According to previous literature, the infection rate of cancer patients was higher than that of the general population [3]. Furthermore, the case-fatality rate of cancer patients infected with SARS-CoV-2 was higher than the overall case-fatality rate of the general population infected with SARS-CoV-2. This indicates that cancer patients are more easily infected with SARS-CoV-2 and have a higher risk of death [4]. Liver injury caused by SARS-CoV-2 could aggravate the condition of patients with hepatocellular carcinoma (HCC) [5].

Liver cancer remains a challenge for global health [6]. It is predicted that by 2025, more than 1 million people will be affected by liver cancer every year [7]. HCC is the most common type of liver tumor, accounting for more than 90% of all liver tumors. Additionally, over 50% of HCC patients are infected by the Hepatitis B virus [8]. Benedicto et al. suggested that in HCC patients with high Neuropilin-1 expression in their liver sinusoidal endothelial cells, their hepatic stellate cells and tumor cells may have a higher chance of SARS-CoV-2 infection [9]. The mortality of cancer patients with COVID-19 is much higher than that in non-cancer patients; however, it is difficult to determine the impact of COVID-19 on HCC because of the limited data on HCC patients with COVID-19 [10]. Microarray data analysis for COVID-19 and the risk factors of HCC is still unknown.

**Fig 1:**
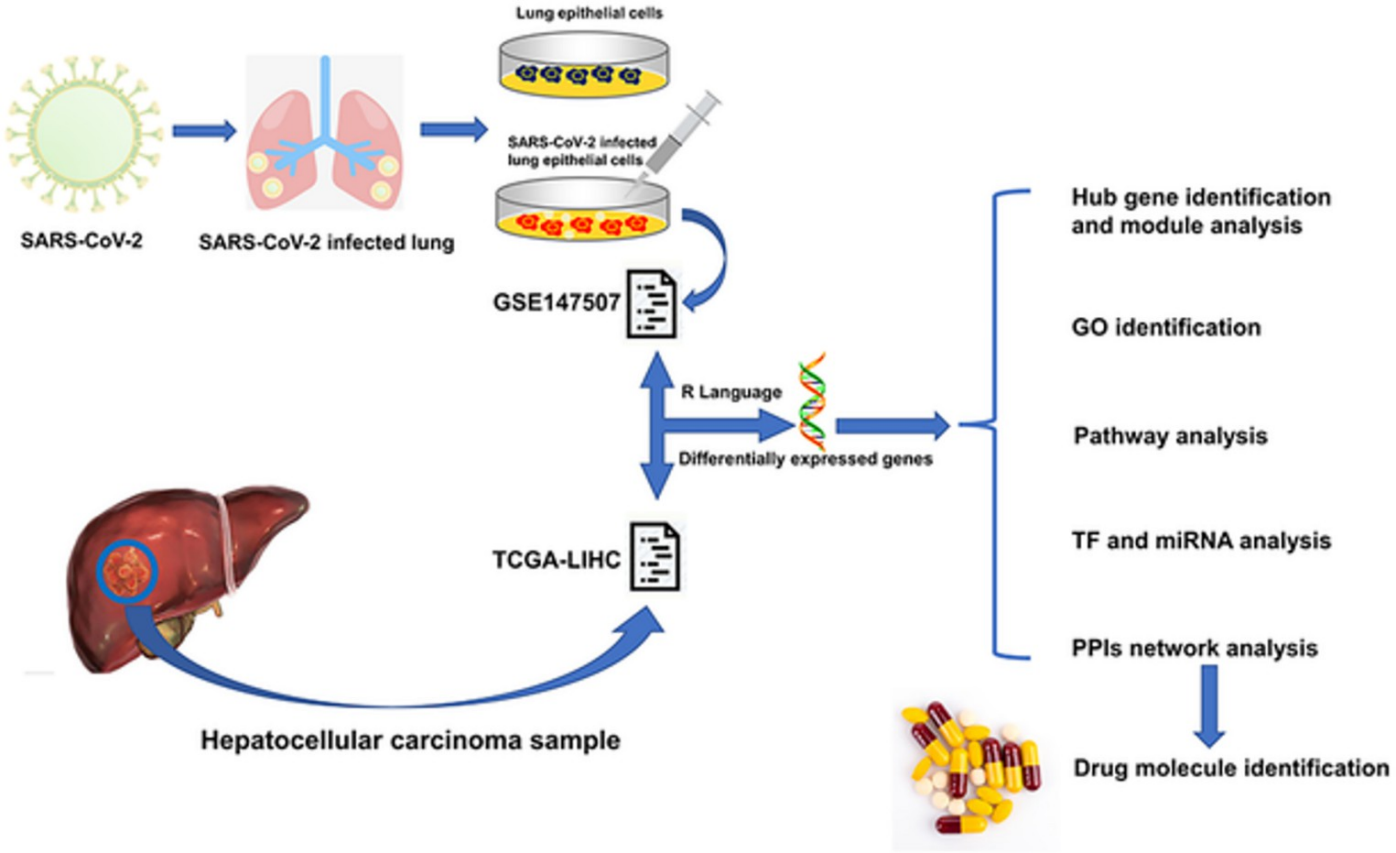
Fundamental workflow for the current study. Two types of samples (Lung epithelial cells, SARS-CoV-2 infected lung epithelial cells) were collected from SARS-CoV-2 infected lung epithelial cells and both are included in the GSE147507 dataset. GSE147507 dataset contains a sample of SARS-CoV-2 infected lung epithelial cells and the TCGA-LIHC dataset contains hepatocellular carcinoma samples. R programming language was used to identify the Common DEGs from both datasets. From the common DEGs, GO identification, KEGG pathway, PPIs network, TF and miRNA analysis, hub gene identification and module analysis were designed and based on those analysis drug molecule identification was performed.

This study attempted to find the common biological pathways and the relationship between HCC and COVID-19. We selected two datasets for our research. The SARS-CoV-2 infection in humans was analyzed using the GSE147507 dataset, while the TCGA-LIHC dataset was used for the gene expression analysis of HCC. First, we identified respectively the differentially expressed genes (DEGs) of GSE147507 and TCGA-LIHC. We then searched common DEGs for COVID-19 and HCC so that we could obtain the basic data for the study. Based on the common DEGs, pathway analysis and gene set enrichment analysis were performed to further understand the biological processes of genome-based expression. Most importantly, we identified hub genes from the common DEGs, a critical step in order to identify relevant effective drug molecules. We designed protein–protein interactions (PPIs) networks to gather hub genes. Fig 1 shows the specific workflow of the present study.

## Materials and methods

### Collection of the dataset

The GSE147507 (https://www.ncbi.nlm.nih.gov/geo/download/?acc=GSE147507) dataset, developed by Blanco-Melo et al. [11], describes the SARS-CoV-2 infection’s transcriptional responses. While the TCGA-LIHC dataset were downloaded from TCGA (https://portal.gdc.cancer.gov/). The GEO database was employed for gene expression analysis under the National Center for Biotechnology Information Platform [12]. The Illumina NextSeq 500 platform was utilized for the GSE147507 dataset for extracted RNA sequence analysis [11]. The COVID-19 dataset (GSE147507) supplies samples consisting of SARS-CoV-2 infection of human lung epithelial and alveolar cells, and the dataset contains 4 samples. We employed the TCGA-LIHC dataset containing 374 tumor samples and 50 paraneoplastic tissue samples. The paraneoplastic tissue samples were used as the control group.

### Identification of DEGs and common genes between COVID-19 and HCC

Pinpointing the common DEGs between the GSE147507 and TCGA-LIHC datasets was the primary task of this study. To discern the DEGs of GSE147507, the researchers used the limma package of the R (V 3.6.3) programming language. The data generated by microarray analysis was retrieved by DESeq2 [13] and the limma package [14]. We set the criteria for considering differences in expression as P values <0.05 and | logFC |>1. Benjamini-Hochberg was employed to control the false discovery rate for both datasets [15]. Common DEGs between the GSE147507 and TCGA-LIHC datasets were identified using the R (V 3.6.3) programming language.

### Gene ontology and pathway findings in terms of gene set enrichment analysis

Gene set enrichment analysis was performed for gene sets with general biological functions and chromosomal locations [16]. For gene product annotation, the GO terminology was utilized, which is divided into three categories: biological processes, molecular functions, and cellular components [17]. The main reason for identifying GO terms is because of the comprehension of molecular activity, cellular action, and the location in the cell where the genes perform their functions. The KEGG pathway is commonly applied to understand metabolic pathways and contains important uses for gene annotation [18]. For a more comprehensive pathway analysis, the WikiPathways [19], Reactome [20] and BioCarta databases were also used. All pathways of common genes identified in the previous step were obtained through the web-based platform Enrichr (https://amp.pharm.mssm.edu/Enrichr/). The Enrichr platform integrates three database resources, WikiPathways, Reactome, and BioCarta. All three databases are capable of performing gene enrichment analysis. For genome-wide genes in experiments, Enrichr provides genomic enrichment analysis on a web-based platform [21].

### Construction of PPIs networks

The common up-regulated and down-regulated DEGs were supplied as input in the Search Tool for Retrieval of Interacting Genes (STRING) (https://string-db.org/). STRING provides interaction-based information based on experiments and predictions, and the interactions generated through the network tool are defined as 3D structures, attachment information, and confidence scores [22]. The confidence score was also utilized for the current PPIs network with a moderate confidence score of 0.400. The confidence score, considered a moderate confidence score, was set using the STRING platform. For the purpose of obtaining a better visual representation of the network and identifying hub genes, the obtained PPIs were analyzed by Cytoscape (https://cytoscape.org/).

### Identification of hub genes and module analysis

The analysis of the PPIs network for the current study was implemented through Cytoscape. The hub genes of the corresponding PPIs networks were indicated using the cytoHubba plugin (http://apps.cytoscape.org/apps/cytohubba). The MCODE (http://apps.cytoscape.org/apps/mcode) plugin was utilized to identify the most profound modules and intensely connection regions in the PPIs network. The identification of highly interconnected parts by MCODE clustering helps to study effective drug design.

### TF-gene interactions

Interactions of TF-genes with identified common DEGs reveal the role TFs play in gene functional pathways and expression levels [23]. The NetworkAnalyst (https://www.networkanalyst.ca/) platform was utilized to identify the interactions of TF-genes with identified common genes. NetworkAnalyst is a synthetic web-based platform for gene expression across multiple species and enables them to be meta-analyzed [24]. The network generated for the TF interaction genes is available from the ENCODE (https://www.encodeproject.org/) database included in the NetworkAnalyst platform.

### TF-miRNA coregulatory network

TF-miRNA co-regulatory interactions were collected from the RegNetwork repository [25], which facilitates the detection of miRNAs and regulatory TFs that regulate DEGs at the post-transcriptional and transcriptional levels. We used NetworkAnalyst to visualize the TF-miRNA co-regulatory network. NetworkAnalyst helped to navigate complicated datasets in the simplest way possible, identifying biological features and functions to derive valid biological hypotheses [26].

### Identification of candidate drugs

Drug molecules were designed based on the COVID-19 and HCC common DEGs using DSigDB, which consists of 22,527 genomes. The Enrichr platform was utilized to identify drug molecules with common DEGs. Data were obtained from the DrugSignatures database (DSigDB). The results of the drug candidates were generated based on P-values. P < 0.05 was set as a statistical criterion. Access to the DSigDB was obtained through the Enrichr platform (https://amp.pharm.mssm.edu/Enrichr/). Enrichr is mainly utilized as an enrichment analysis platform, providing numerous visual details of the collective function of the genes provided for input [27].

## Results

### Identification of common DEGs between COVID-19 and HCC

**Fig 2:**
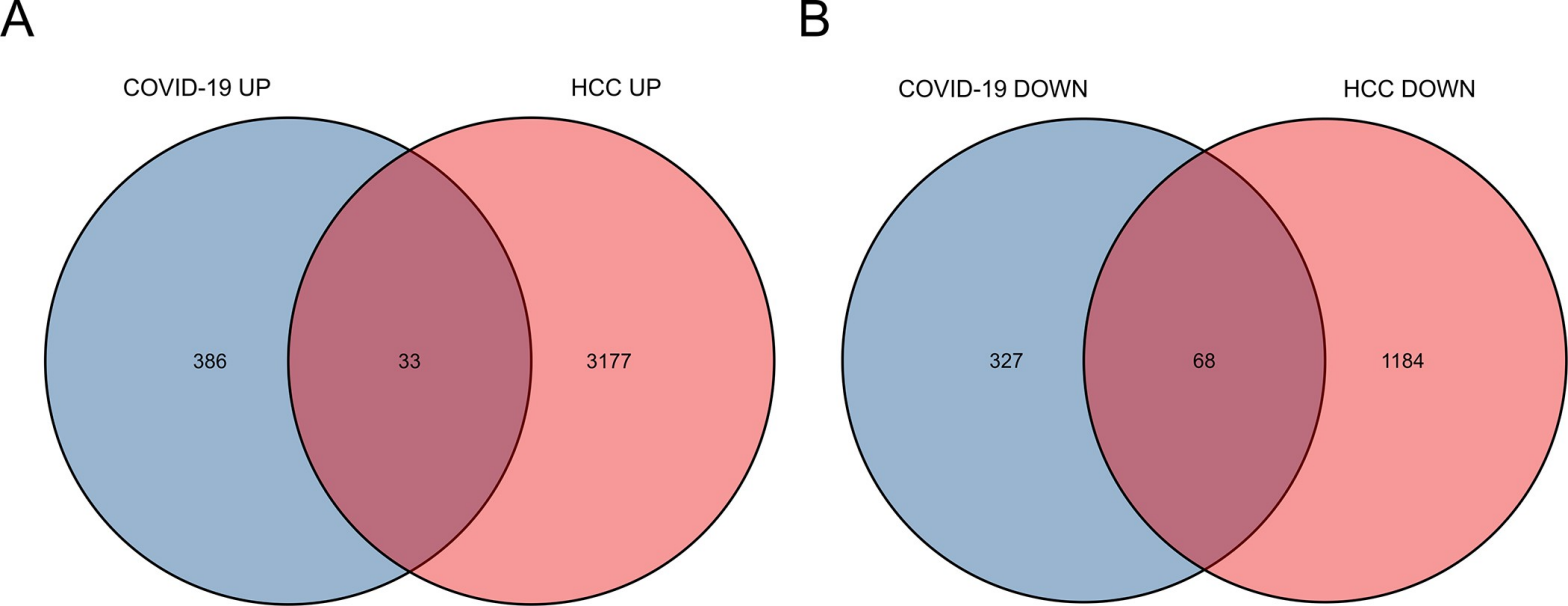
Common differentially expressed genes represented by Venn diagrams. 33 commonly differentially up-regulated expressed genes and 68 commonly differentially down-regulated expressed genes were identified from 814 differentially expressed genes in SARS-CoV-2 infection and 4462 differentially expressed genes in HCC patients.

The GSE147507 dataset was utilized to identify DEGs for COVID-19. 814 DEGs were identified, including 419 up-regulated genes and 395 down-regulated genes. For the HCC dataset, TCGA-LIHC was utilized to identify a total of 4,462 DEGs, of which 3,210 genes were up-regulated and 1,252 genes were down-regulated. As shown in Fig 2, combining the results of the above analysis, we identified 33 DEGs with common up-regulated expression and 68 DEGs with common down-regulated expression. These two sets of the DEGs were used to complete further analysis.

### Gene ontology and pathway findings in terms of gene set enrichment analysis

**Fig 3:**
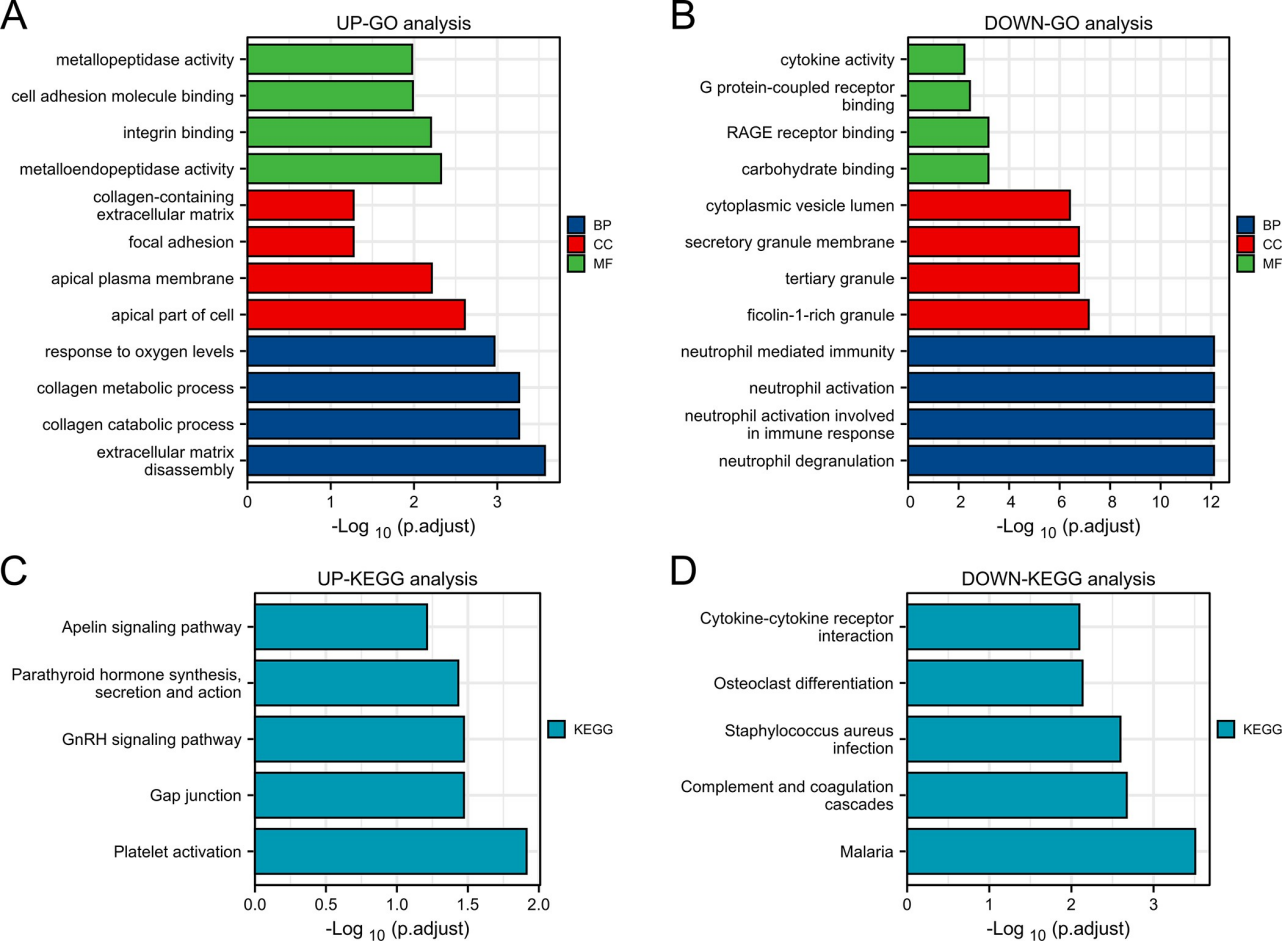
(A, B) Identification results of biological processes, cellular components and molecular functions related to GO terms based on a composite score. The higher the enrichment score, the higher the number of genes involved in a given ontology. (C, D) Identification of pathway analysis results by KEGG. The results of pathway term identification by composite score.

**Fig 4:**
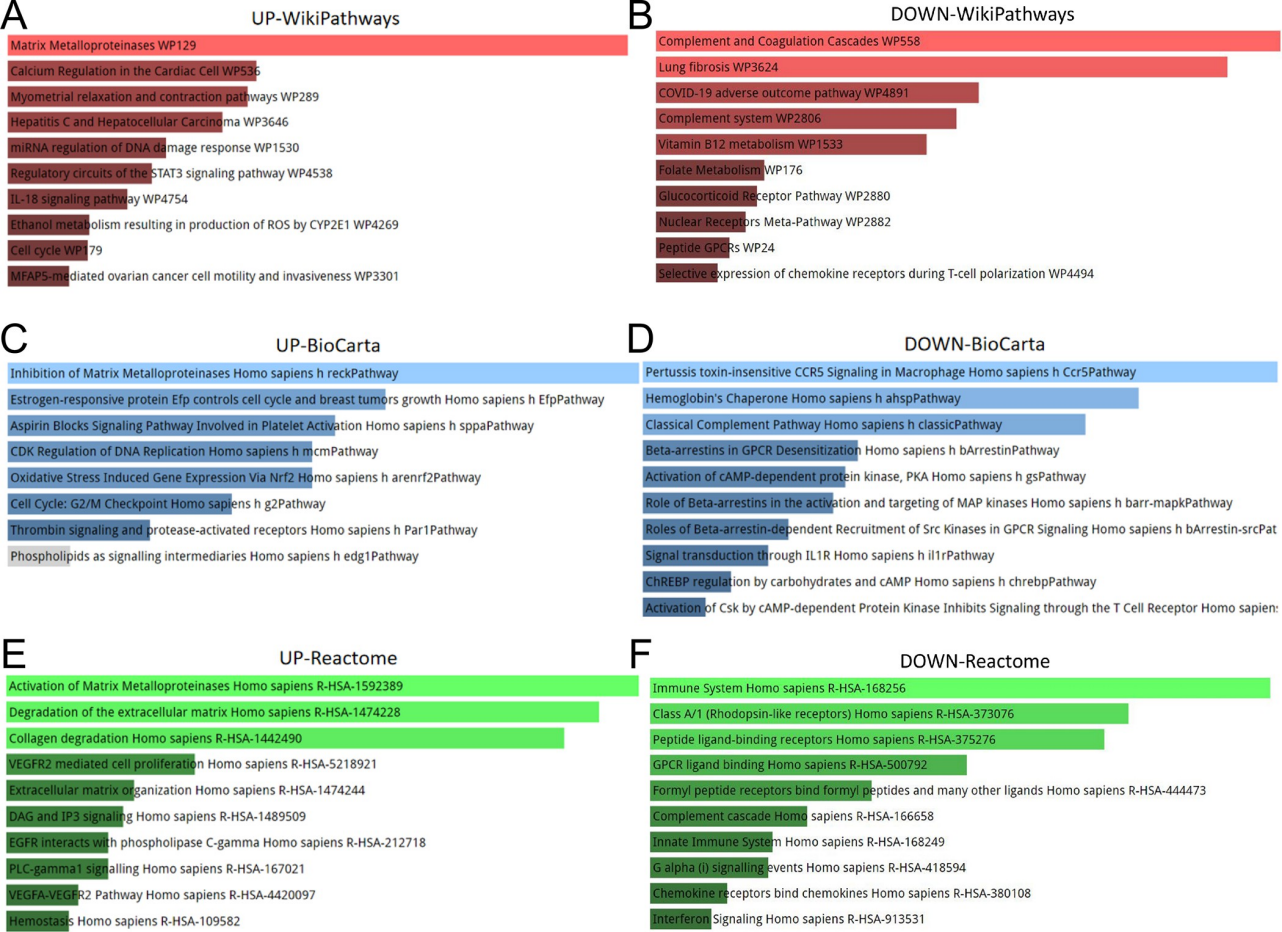
Pathway analysis was performed by WikiPathways, BioCarta and Reactome for result identification: (A, B) WikiPathways, (C, D) BioCarta and (E, F) Reactome.

The current study analyzes GO terms and KEGG pathway for the 33 DEGs with common up-regulated expression and 68 DEGs with common down-regulated expression. The three best known GO terms include biological processes, molecular functions, and cellular components. Our studies illustrate the top GO terms for each subsection (biological processes, cellular components, and molecular functions), as shown in Table 1. The data in Table 1 demonstrate that for biological processes, common 33 DEGs with common up-regulated expression are highly enhanced in extracellular matrix disassembly and collagen catabolic process. 68 DEGs with common down-regulated expression are highly enhanced in neutrophil degranulation and neutrophil activation involved in immune response. Data from the molecular function subsection suggest that metalloendopeptidase activity is well correlated with 33 common up-regulated genes. Correspondingly, carbohydrate binding is well correlated with 68 DEGs with common down-regulated expression. Cellular component studies revealed significant involvement of apical part of cell and ficolin-1-rich granule respectively in common up-regulated and down-regulated DEGs. Table 2 shows the interaction of the complement and coagulation cascades with most genes according to the KEGG pathway database. The information obtained from Table 2 indicates the interaction of the platelet activation pathway and the malaria pathway with most genes according to the KEGG pathway database. Fig 3 shows the visualization results of the GO and KEGG analysis. WikiPathways, Reactome and BioCarta pathway analyses are summarized in Table 3. Fig 4 illustrates the results of the pathway analysis from the diverse pathway databases, respectively.

**Table 1:** GO analysis of common up-regulated DEGs and common down-regulated DEGs among COVID-19 and HCC.

ONTOLOGY	ID	Description	pvalue	p.adjust
UP-GO biological process	GO:0022617	extracellular matrix disassembly	2.34e-07	2.68e-04
	GO:0030574	collagen catabolic process	1.20e-06	5.47e-04
	GO:0032963	collagen metabolic process	1.43e-06	5.47e-04
	GO:0070482	response to oxygen levels	3.77e-06	0.001
UP-GO Cellular Component	GO:0045177	apical part of cell	2.56e-05	0.002
	GO:0016324	apical plasma membrane	1.27e-04	0.006
	GO:0005925	focal adhesion	0.004	0.053
	GO:0062023	collagen-containing extracellular matrix	0.004	0.053
UP-GO Molecular Function	GO:0004222	metalloendopeptidase activity	3.02e-05	0.005
	GO:0005178	integrin binding	7.96e-05	0.006
	GO:0050839	cell adhesion molecule binding	1.97e-04	0.010
	GO:0008237	metallopeptidase activity	2.68e-04	0.011
DOWN-GO biological process	GO:0043312	neutrophil degranulation	1.13e-15	7.71e-13
	GO:0002283	neutrophil activation involved in immune response	1.27e-15	7.71e-13
	GO:0042119	neutrophil activation	1.84e-15	7.71e-13
	GO:0002446	neutrophil mediated immunity	1.91e-15	7.71e-13
DOWN-GO Cellular Component	GO:0101002	ficolin-1-rich granule	6.47e-10	7.05e-08
	GO:0070820	tertiary granule	4.33e-09	1.72e-07
	GO:0030667	secretory granule membrane	4.74e-09	1.72e-07
	GO:0060205	cytoplasmic vesicle lumen	1.74e-08	3.92e-07
DOWN-GO Molecular Function	GO:0030246	carbohydrate binding	4.51e-06	6.59e-04
	GO:0050786	RAGE receptor binding	6.62e-06	6.59e-04
	GO:0001664	G protein-coupled receptor binding	5.38e-05	0.004
	GO:0005125	cytokine activity	1.19e-04	0.006

Note: Top 4 terms of each category are listed. UP: common up-regulated DEGs; DOWN: common down-regulated DEGs

**Table 2:** KEGG analysis of common up-regulated DEGs and common down-regulated DEGs among COVID-19 and HCC.

ONTOLOGY	ID	Description	pvalue	p.adjust
UP-KEGG	hsa04611	Platelet activation	1.08e-04	0.012
	hsa04540	Gap junction	7.61e-04	0.034
	hsa04912	GnRH signaling pathway	8.94e-04	0.034
	hsa04928	Parathyroid hormone synthesis, secretion and action	0.001	0.037
	hsa04371	Apelin signaling pathway	0.003	0.061
DOWN-KEGG	hsa05144	Malaria	4.14e-06	3.11e-04
	hsa04610	Complement and coagulation cascades	5.65e-05	0.002
	hsa05150	Staphylococcus aureus infection	1.01e-04	0.003
	hsa04380	Osteoclast differentiation	3.90e-04	0.007
	hsa04060	Cytokine-cytokine receptor interaction	5.34e-04	0.008

Note: Top 5 terms of each category are listed. UP: common up-regulated DEGs; DOWN: common down-regulated DEGs

**Table 3:** Top 9 pathways from WikiPathways, BioCarta and Reactome databases for common up-regulated DEGs and common down-regulated DEGs among COVID-19 and HCC.

Databases	Pathways	P-value	Adjusted p-value
UP-WikiPathways	Matrix Metalloproteinases WP129	0.00001612	0.001451
	Ethanol metabolism resulting in production of ROS by CYP2E1 WP4269	0.01638	0.1301
	MFAP5-mediated ovarian cancer cell motility and invasiveness WP3301	0.02125	0.1301
	Osteoblast Signaling WP322	0.02286	0.1301
	Major receptors targeted by epinephrine and norepinephrine WP4589	0.02447	0.1301
	Biomarkers for pyrimidine metabolism disorders WP4584	0.02447	0.1301
	GPR40 Pathway WP3958	0.02447	0.1301
	Hepatitis C and Hepatocellular Carcinoma WP3646	0.002958	0.06655
	Airway smooth muscle cell contraction WP4962	0.02769	0.1301
DOWN-WikiPathways	COVID-19 adverse outcome pathway WP4891	0.00001661	0.0005444
	Complement and Coagulation Cascades WP558	0.000001559	0.0001298
	Lung fibrosis WP3624	0.00000236	0.0001298
	Selective expression of chemokine receptors during T-cell polarization WP4494	0.0001289	0.001418
	Vitamin B12 metabolism WP1533	0.00002501	0.0005503
	Platelet-mediated interactions with vascular and circulating cells WP4462	0.001499	0.01268
	Activation of NLRP3 Inflammasome by SARS-CoV-2 WP4876	0.02356	0.07623
	Nanomaterial-induced inflammasome activation WP3890	0.02356	0.07623
	Hfe effect on hepcidin production WP3924	0.02356	0.07623
UP-BioCarta	Inhibition of Matrix Metalloproteinases Homo sapiens h reckPathway	0.01313	0.04688
	Estrogen-responsive protein Efp controls cell cycle and breast tumors growth Homo sapiens h EfpPathway	0.02447	0.04688
	Aspirin Blocks Signaling Pathway Involved in Platelet Activation Homo sapiens h sppaPathway	0.02769	0.04688
	CDK Regulation of DNA Replication Homo sapiens h mcmPathway	0.0293	0.04688
	Oxidative Stress Induced Gene Expression Via Nrf2 Homo sapiens h arenrf2Pathway	0.0293	0.04688
	Cell Cycle: G2/M Checkpoint Homo sapiens h g2Pathway	0.0357	0.04759
	Thrombin signaling and protease-activated receptors Homo sapiens h Par1Pathway	0.04363	0.04987
	Phospholipids as signalling intermediaries Homo sapiens h edg1Pathway	0.05308	0.05308
	Inhibition of Matrix Metalloproteinases Homo sapiens h reckPathway	0.01313	0.04688
DOWN-BioCarta	Pertussis toxin-insensitive CCR5 Signaling in Macrophage Homo sapiens h Ccr5Pathway	0.0004038	0.008523
	Hemoglobin’s Chaperone Homo sapiens h ahspPathway	0.0008672	0.008523
	Classical Complement Pathway Homo sapiens h classicPathway	0.001162	0.008523
	Beta-arrestins in GPCR Desensitization Homo sapiens h bArrestinPathway	0.004067	0.01708
	Activation of cAMP-dependent protein kinase, PKA Homo sapiens h gsPathway	0.004358	0.01708
	Role of Beta-arrestins in the activation and targeting of MAP kinases Homo sapiens h barr-mapkPathway	0.004659	0.01708
	Alternative Complement Pathway Homo sapiens h alternativePathway	0.03349	0.05394
	G-Protein Signaling Through Tubby Proteins Homo sapiens h tubbyPathway	0.03349	0.05394
	Regulators of Bone Mineralization Homo sapiens h npp1Pathway	0.03678	0.05394
UP-Reactome	Activation of Matrix Metalloproteinases Homo sapiens R-HSA-1592389	0.00001965	0.002813
	Collagen degradation Homo sapiens R-HSA-1442490	0.00003592	0.002813
	Defective CHST3 causes SEDCJD Homo sapiens R-HSA-3595172	0.01149	0.06431
	Defective CHSY1 causes TPBS Homo sapiens R-HSA-3595177	0.01149	0.06431
	Defective CHST14 causes EDS, musculocontractural type Homo sapiens R-HSA-3595174	0.01149	0.06431
	DAG and IP3 signaling Homo sapiens R-HSA-1489509	0.00127	0.0218
	Creatine metabolism Homo sapiens R-HSA-71288	0.01638	0.08369
	GP1b-IX-V activation signalling Homo sapiens R-HSA-430116	0.01638	0.08369
	Adenylate cyclase activating pathway Homo sapiens R-HSA-170660	0.01638	0.08369
DOWN-Reactome	Formyl peptide receptors bind formyl peptides and many other ligands Homo sapiens R-HSA-444473	0.000002079	0.00005781
	DEx/H-box helicases activate type I IFN and inflammatory cytokines production Homo sapiens R-HSA-3134963	0.0008672	0.007534
	Advanced glycosylation endproduct receptor signaling Homo sapiens R-HSA-879415	0.0008672	0.007534
	Regulation of Complement cascade Homo sapiens R-HSA-977606	0.00009241	0.00107
	Ficolins bind to repetitive carbohydrate structures on the target cell surface Homo sapiens R-HSA-2855086	0.01689	0.07572
	Scavenging by Class B Receptors Homo sapiens R-HSA-3000471	0.01689	0.07572
	Peptide ligand-binding receptors Homo sapiens R-HSA-375276	1.82E-08	8.41E-07
	Complement cascade Homo sapiens R-HSA-166658	0.000007721	0.0001789
	Chemokine receptors bind chemokines Homo sapiens R-HSA-380108	0.00003929	0.0006068

### PPIs network to identify hub genes and module analysis

**Fig 5:**
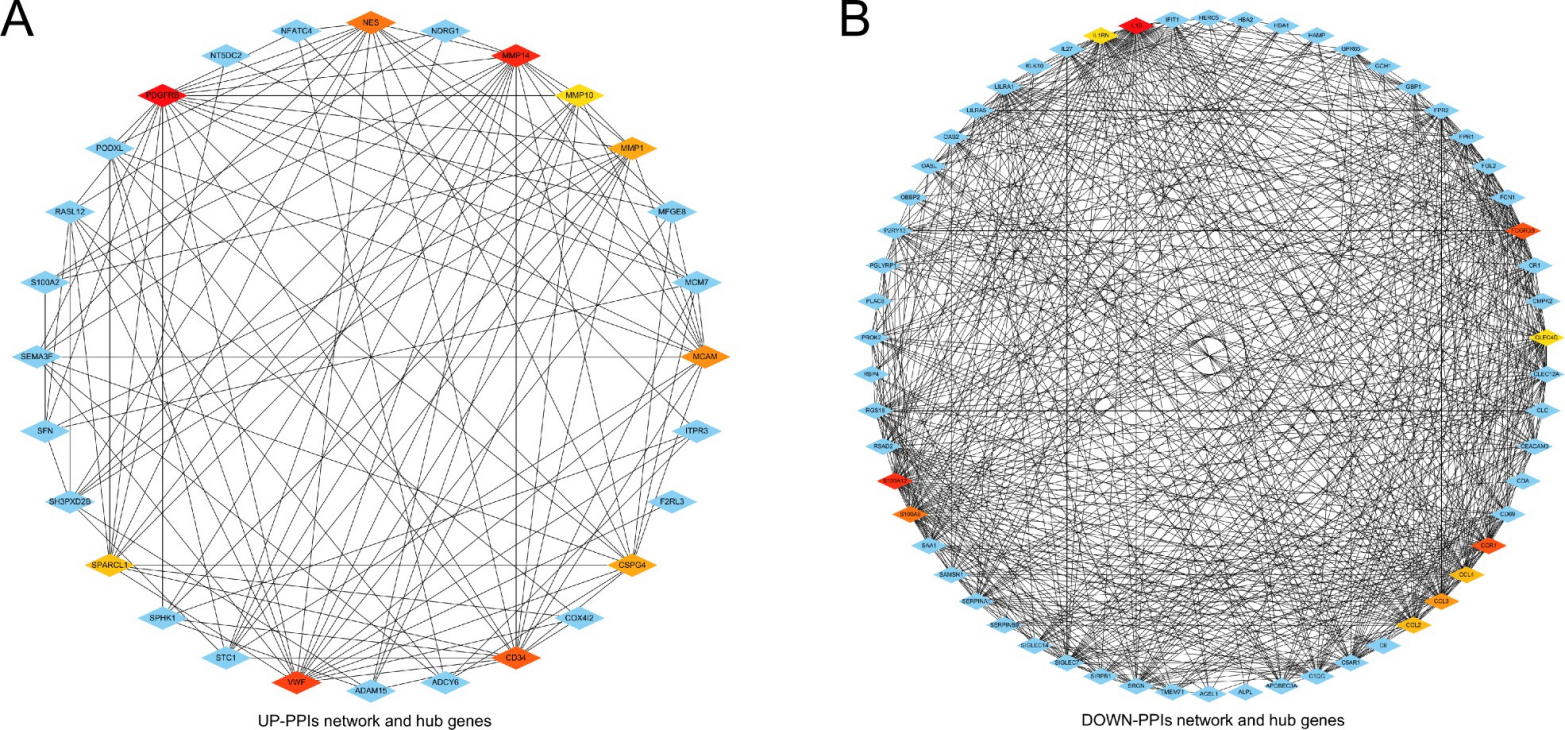
(A) The network of protein-protein interactions (PPIs) for identifying common differentially up-regulated expressed genes common to both diseases (COVID-19 and HCC). Nodes indicate common differentially expressed genes and edges specify the interconnection between two genes. The analyzed network has 28 nodes and 118 edges. Hub genes were detected from a network of PPIs with common differentially expressed genes. The 5 genes highlighted are PDGFRB, MMP14, VWF, MMP1 and NES. (B) The network of PPIs for identifying common differentially down-regulated expressed genes common to both diseases (COVID-19 and HCC). The analyzed network has 58 nodes and 645 edges. The 5 hub genes are IL1B, S100A12, FCGR3B, CCR1 and S100A8.

The PPIs network was created for further analysis of this study, including the hub gene assay used to identify drug molecules for COVID-19 and HCC. Ultimately, the results of the PPIs network were connected, and the PPIs analysis was proposed to build the drug compounds at the center of this study. The PPIs network of common up-regulated DEGs (UP-PPIs network) contains 28 nodes and 118 edges, as shown in Fig 5A. The PPIs network of common down-regulated DEGs (DOWN-PPIs network) contains 58 nodes and 645 edges, as shown in Fig 5B.

### Identification of hub genes and module analysis for suggesting therapeutic solutions

**Fig 6:**
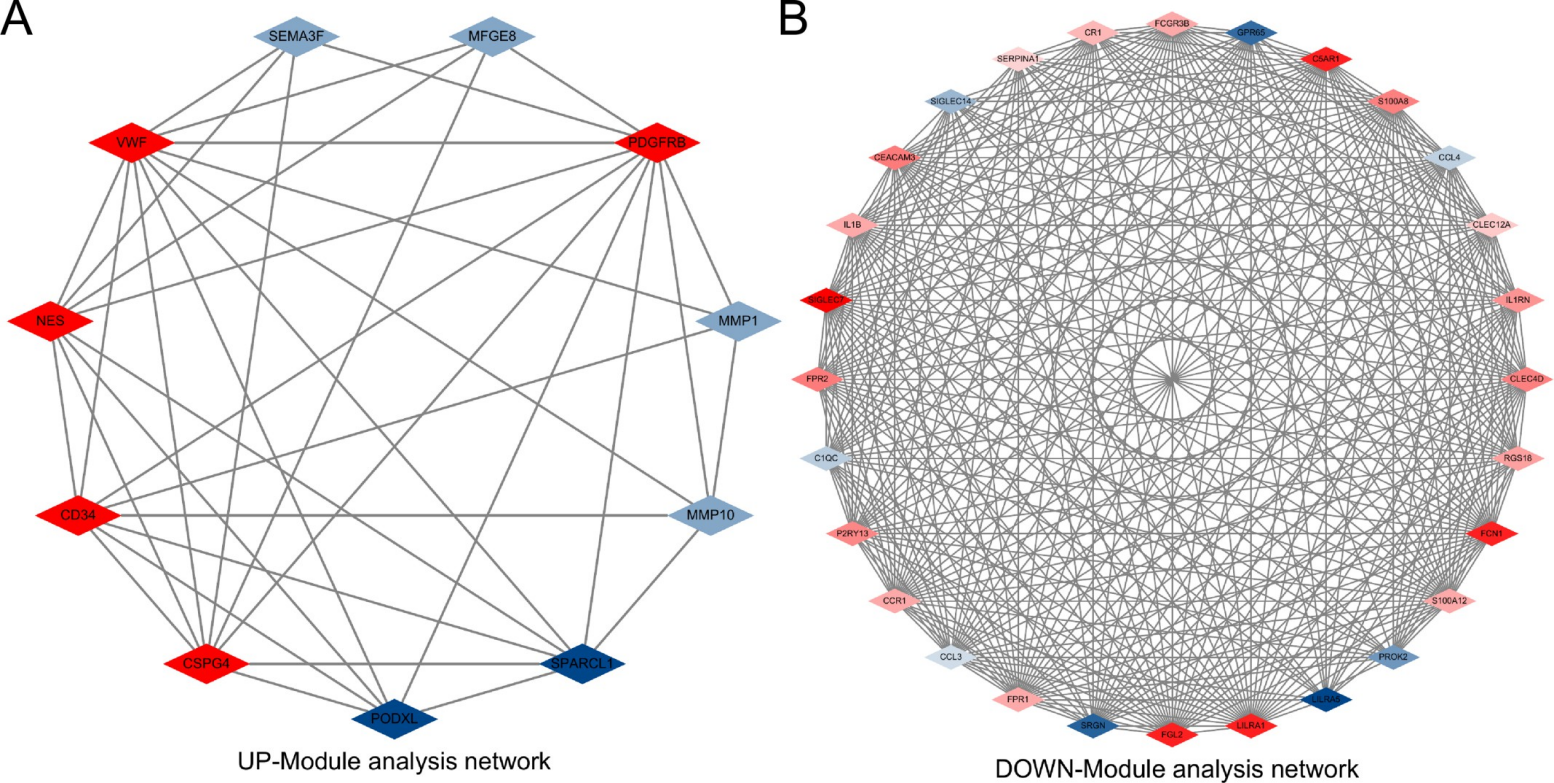
The module analysis network was obtained from the PPIs network in Fig 5. PDGFRB, VWF and SIGLEC7 are highlighted in red, as these 3 hub nodes are common between GSE147507 and TCGA-LIHC. This network represents the highly interconnected region of the PPIs network.

To track hub genes from the network of PPIs highlighted in Fig 5, cytohubba, a plug-in for the Cytoscape software, was utilized. Hub genes were sorted by their degree values, which indicated the number of gene interactions in the PPIs network. In the UP-PPIs network, the top 10 identified hub genes were *PDGFRB*, *MMP14*, *VWF*, *CD34*, *NES*, *MCAM*, *CSPG4*, *MMP1*, *SPARCL1* and *MMP10*. In the DOWN-PPIs network, the top 10 identified hub genes were *IL1B*, *S100A12*, *FCGR3B*, *CCR1*, *S100A8*, *CCL3*, *CCL2*, *CCL4*, *CLEC4D* and *LILRA1*. Fig 5 shows the interaction of the hub proteins with other proteins in the PPIs network. Highly dense modules were designed from the PPIs network using The Molecular Complex Detection (MCODE), another plug-in for the Cytoscape software. *PDGFRB* and *VWF* are two genes highlighted in the module network of common up-regulated DEGs (UP-Module analysis network). *SIGLEC7* is the gene highlighted in the module network of common down-regulated DEGs (DOWN-Module analysis network). Module analysis is shown in Fig 6. The UP-Module analysis network contains 11 nodes and 37 edges. The DOWN-Module analysis network contains 28 nodes and 331 edges. Topological analysis was performed using cytohubba to identify the hub genes. The results of the topological analysis are shown in Table 4.

**Table 4:** Topological results for the first 10 hub genes.

Network	Hub gene	Degree	Stress	Closeness	Betweenness	Eccentricity
UP-PPIs	PDGFRB	18	344	23	100.03175	0.5
	MMP14	17	286	22.5	88.55397	0.5
	VWF	16	326	22	113.31508	0.5
	CD34	15	168	21.33333	45.70714	0.33333
	NES	14	168	21	40.75833	0.5
	MCAM	13	112	20.5	21.62063	0.5
	CSPG4	12	126	20	28.90952	0.5
	MMP1	12	112	19.5	28.31548	0.33333
	SPARCL1	11	152	19.33333	43.15516	0.33333
	MMP10	10	52	18.66667	9.63571	0.33333
DOWN-PPIs	IL1B	49	2510	55.33333	500.22298	0.33333
	S100A12	45	1438	53.33333	199.29362	0.33333
	FCGR3B	42	1316	51.83333	198.22844	0.33333
	CCR1	42	1070	51.83333	121.50458	0.33333
	S100A8	41	1178	51.33333	177.54056	0.33333
	CCL3	37	668	49.33333	71.37717	0.33333
	CCL2	36	1106	48.83333	156.4397	0.33333
	CCL4	36	634	48.83333	64.33257	0.33333
	CLEC4D	35	578	48.16667	46.80746	0.33333
	LILRA1	35	590	48.16667	53.01227	0.33333

### Transcription factor-gene interactions

**Fig 7:**
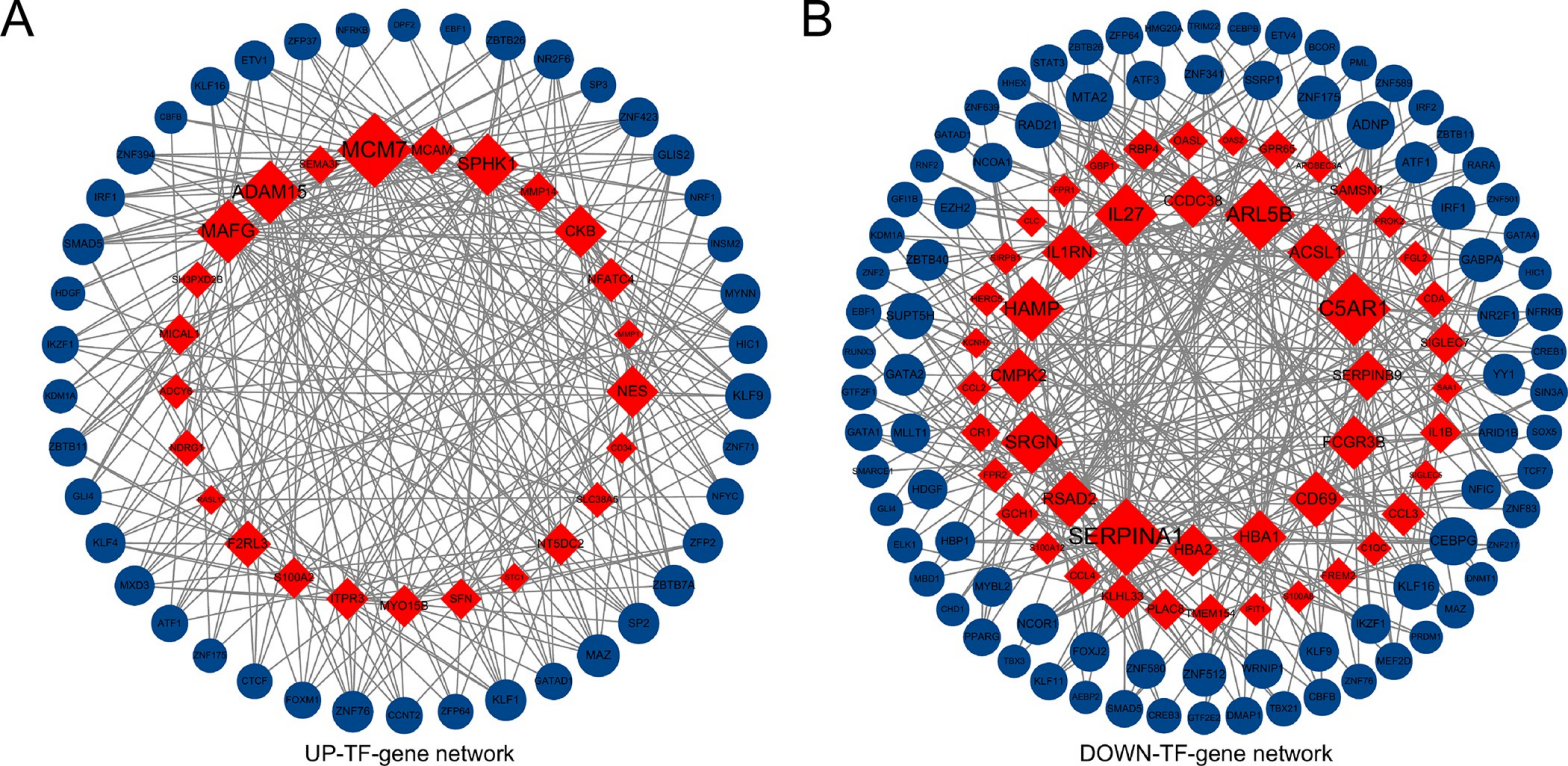
Network of TF-genes interacting with common differentially expressed genes. The highlighted red nodes represent common genes and the other nodes represent TF-genes.

Transcription factor- (TF) gene interactions were gathered using NetworkAnalyst. For the common up-regulated and down-regulated DEGs, the TF-genes were characterized. The interaction of the TF regulators with the common up-regulated and down-regulated DEGs is shown in Fig 7. For the common up-regulated DEGs, the network consists of 67 nodes and 278 edges (UP-TF-gene network). The network includes a total of 42 TF-genes. *MCM7* is mainly regulated by 33 TF-genes, *ADAM17* is mainly regulated by 25 TF-genes, and *MAFG* is mainly regulated by 25 TF-genes. For the common down-regulated DEGs, the network consists of 135 nodes and 358 edges (DOWN-TF-gene network). The network includes a total of 86 TF-genes. *SERPINA1* is mainly regulated by 23 TF-genes, *C5AR1* is mainly regulated by 21 TF-genes, and *ARL5B* is mainly regulated by 20 TF-genes. These TF-genes moderate the network of more than one common DEG, which demonstrates the high interaction of TF-genes with the common up-regulated and down-regulated DEGs. Fig 7 illustrates the TF-gene interaction network.

### TF-microRNA coregulatory network

**Fig 8:**
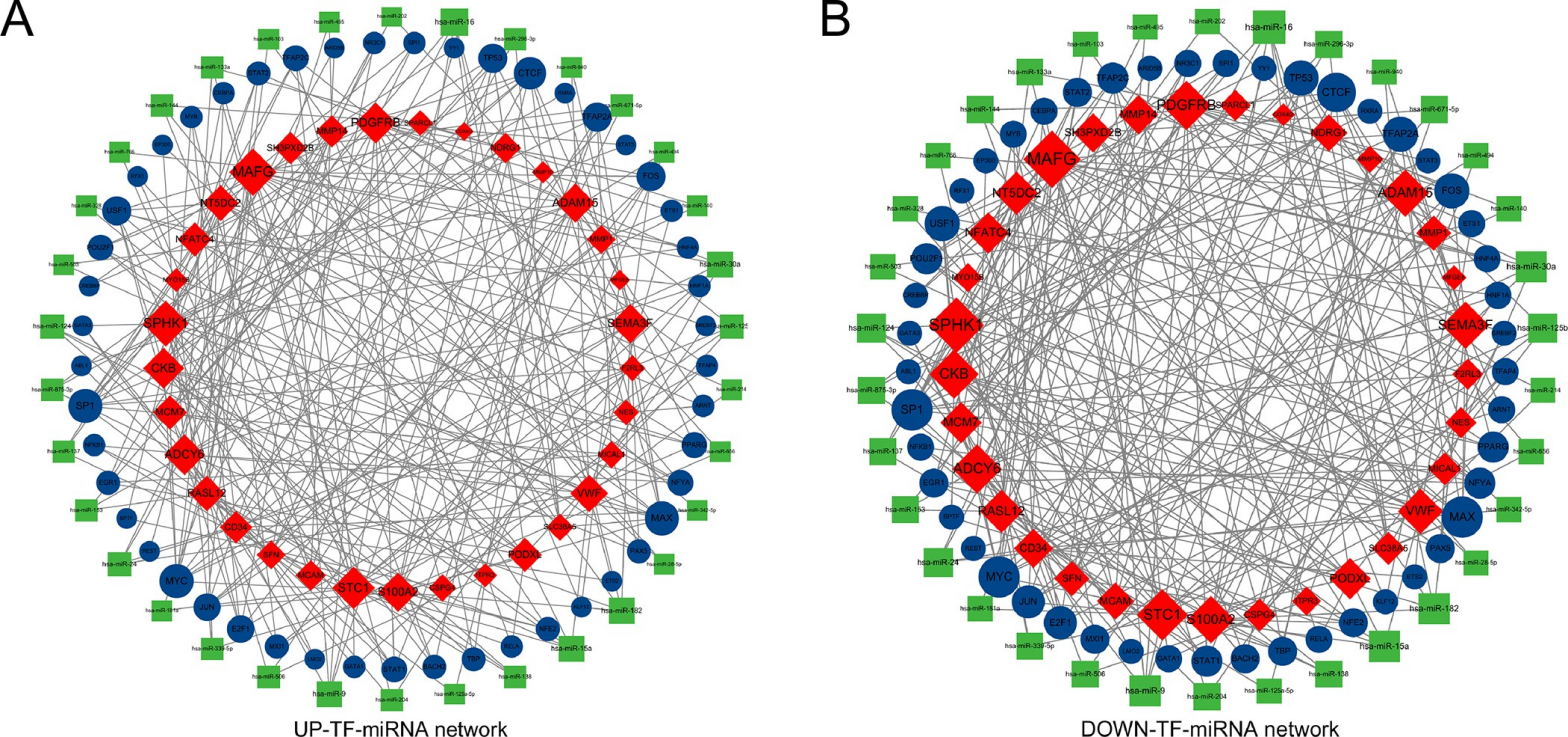
The network presents a TF-miRNA co-regulatory network. Red, orange and yellow nodes are differentially expressed genes, green nodes indicate miRNAs and blue nodes indicate TF-genes.

TF-microRNA (miRNA) coregulatory network was produced using NetworkAnalyst. Analysis of the TF-miRNA co-regulatory network and provides the interaction of miRNA and TFs with the common up-regulated and down-regulated DEGs. This interaction may be responsible for regulating the expression of DEGs. For the common up-regulated DEGs, the network created for the TF-miRNA co-regulatory network includes 116 nodes and 299 edges (UP-TF-miRNA network). Among all miRNAs involved in regulating genes, the largest number of regulated genes was has-miR-16, which was involved in regulating 6 genes. For the common down-regulated DEGs, the network created for the TF-miRNA co-regulatory network includes 191 nodes and 452 edges (DOWN-TF-miRNA network). Similarly, the largest number of regulated genes in this network is has-miR-9, which is involved in the regulation of 7 genes. Fig 8 illustrates the TF-miRNA co-regulatory network.

### Identification of candidate drugs

This study aimed to integrate the treatment of COVID-19 and HCC. According to the DSigDB, drug molecules were derived from the 33 common up-regulated DEGs and 68 common down-regulated DEGs. Among all drug candidates based on adjusted P-values (P < 0.05), the current study highlights the top ten important drugs. Ilomastat TTD 00008545, CGS-27023A TTD 00002801, CHEMBL475540 TTD 00006054, LAMININ BOSS, Plasmasteril BOSS, Ethylene dimethacrylate BOSS, 5194442 MCF7 UP, 9001-31-4 BOSS, fluphenazine PC3 UP and norcyclobenzaprine PC3 UP are the peak candidates for COVID-19 and HCC according to common up-regulated DEGs. Palmatine CTD 00000225, betamethasone CTD 00005504, fludrocortisone CTD 00005975, suloctidil HL60 UP, Modrasone CTD 00001031, Roflumilast CTD 00003916, acetohexamide PC3 UP, alexidine CTD 00000048, Antimycin A CTD 00005427 and dequalinium CTD 00005770 are the best candidates for COVID-19 and HCC based on common down-regulated DEGs. For the common up-regulated DEGs, the analysis showed that Ilomastat TTD 00008545, CGS-27023A TTD 00002801 and CHEMBL475540 TTD 00006054 were the top 3 drug molecules that interacted with the most genes. For the common down-regulated DEGs, the analysis showed that Palmatine CTD 00000225, betamethasone CTD 00005504 and fludrocortisone CTD 00005975 were the top 3 drug molecules that interacted with the most genes. Since these characteristic drugs were detected against common DEGs, these drugs represent common drugs for COVID-19 and HCC. Tables 5 and 6 indicate the drug candidates for the common DEGs in the DSigDB.

**Table 5:** List of the suggested drugs for UP-DEGs of HCC with COVID-19.

Name of drugs	P-value	Adjusted p-value	gene
Ilomastat TTD 00008545	0.0001725	0.01056	MMP14, MMP1
CGS-27023A TTD 00002801	0.0002036	0.01056	MMP14, MMP1
CHEMBL475540 TTD 00006054	0.0002373	0.01161	MMP14, MMP10
LAMININ BOSS	8.64E-10	7.61E-07	PDGFRB, MMP14
Plasmasteril BOSS	0.0004924	0.01735	VWF, CD34
Ethylene dimethacrylate BOSS	0.000001365	0.0003252	VWF, MMP1
5194442 MCF7 UP	0.0007734	0.02129	MAFG. NDRG1
9001-31-4 BOSS	0.000002652	0.0004673	MMP14, VWF;
fluphenazine PC3 UP	0.0009715	0.02195	MMP1, NDRG1
norcyclobenzaprine PC3 UP	0.001042	0.02296	MMP1, NDRG1

**Table 6:** List of the suggested drugs for DOWN-DEGs of HCC with COVID-19.

Name of drugs	P-value	Adjusted p-value	gene
Palmatine CTD 00000225	2.81E-07	0.00007013	SLC5A7, CCL4;
betamethasone CTD 00005504	2.81E-07	0.00007013	IL1B, CCL4;
fludrocortisone CTD 00005975	0.000006082	0.0007	CCL4, CCL3;
suloctidil HL60 UP	1.36E-15	2.04E-12	CCR1, IL1RN;
Modrasone CTD 00001031	0.00000809	0.0008069	CCL4, CCL3
Roflumilast CTD 00003916	0.00001049	0.000981	CCL4, CCL3;
acetohexamide PC3 UP	0.000001031	0.0002202	RSAD2, OAS2;
alexidine CTD 00000048	0.00002039	0.001525	CCL4, CCL3;
Antimycin A CTD 00005427	0.000001727	0.0003073	IL1B, CCL4;
dequalinium CTD 00005770	0.00003503	0.002096	CCL4, CCL3;

## Discussion

There are no previous studies showing that having liver cancer is a risk factor for COVID-19. When a patient has liver cancer or even advanced liver cancer, the normal physiological functions of the liver are disrupted, and many critical proteins cannot be synthesized. This condition may promote the development of COVID-19. This study contributes to the narrative Bioinformatics curriculum for the meaningful analysis of SARS-CoV-2 affected lung epithelial and alveolar samples and HCC affected human liver tissue. Bioinformatics-related methods were used for this study to screen 814 and 4,462 DEGs from GSE147507 and TCGA-LIHC, respectively. To establish relationships and detect drug candidates based on COVID-19 and HCC, common DEGs were identified between the GSE147507 and TCGA-LIHC datasets. A total of 33 common up-regulated DEGs and 68 common down-regulated DEGs were identified. Next, we continued to analyze GO, the pathway, PPIs networks, TF-gene interactions, the TF-miRNA coregulatory network, and drug candidate assays.

33 common up-regulated DEGs and 68 common down-regulated DEGs were identified for detecting GO terms. GO terms were selected based on P-values. Extracellular matrix disassembly, neutrophil degranulation, metalloendopeptidase activity, carbohydrate binding, apical part of cell and ficolin-1-rich granule were the most important GO terms. Extracellular matrix breakdown is strongly associated with the pathogenic process of COVID-19. SARS-CoV-2 induces an inflammatory response at the blood-gas barrier, causing the breakdown of adherens junctions and tight junctions between endothelial cells, leading to the collapse of the blood-gas barrier and ultimately to hypoxia [28]. Neutrophils play an important role in the course of COVID19 infection. Increased hyperactivated degranulated neutrophils in alveolar lavage fluid of patients with neoconiosis compared to influenza [29]. Carbohydrate binding leads to insulin resistance, the thereby being involved in the pathogenesis of diabetes, COVID-19 may also predispose infected individuals to hyperglycemia [30, 31]. The top ranked GO terms according to cellular component are the apical portion of the cell and the paclitaxel-1 enriched granules.

The KEGG pathway of the 33 common up-regulated DEGs and 68 common down-regulated DEGs was identified, as similar pathways were found for COVID-19 and HCC. The top 2 KEGG pathways included platelet activation and Malaria. Increased platelet activation and platelet-monocyte aggregate formation can be observed in patients with severe COVID-19, while platelet activation is associated with the severity of COVID-19 and mortality [32]. Platelet activation is increased in steatosis to nonalcoholic steatohepatitis (NASH), and antiplatelet therapy may prevent the development of NASH and subsequent HCC [33]. A study by Wang et al. found that malaria effectively inhibited HCC progression and prolonged survival time in tumor-bearing mice [34]. Concurrently, the results of WikiPathways indicated that the most interactive gene pathway was matrix metalloproteinases and COVID-19 adverse outcome pathway. A study by Petito et al. also suggested that plasma matrix metalloproteinase 9 was significantly increased in COVID-19 patients [35]. The results from the Reactome and BioCarta pathway also implicated that the most critical pathway is the activation of matrix metalloproteinases and the binding of formylated peptides by formylated peptide receptors.

Because hub gene detection, module analysis, and drug identification depend entirely on the PPIs network, the PPIs network analysis is the most remarkable part of the study. The PPIs analysis was also generated for 33 common up-regulated DEGs and 68 common down-regulated DEGs. According to the PPIs network the *PDGFRB*, *MMP14*, *VWF*, *IL1B*, *S100A12* and *FCGR3B* genes were declared central genes due to their high interaction rate or degree values. High expression of *PDGFRB* and *MMP14* is associated with poor prognosis of HCC [36–38]. VWF has also been mentioned several times as a biomarker to predict the prognosis of liver cancer [33, 39, 40]. VWF is involved in the formation of microvascular thrombi during COVID19 pathogenesis [41]. The inflammatory cytokine IL-1β was proven to be responsible for the induction of PD-L1 expression, further mediating immune escape from HCC [42]. Del Valle et al. revealed low levels of IL-1β expression in the serum of COVID-19 patients [43]. S100A12 was low-expressed in HCC tissues, and lower expression of S100A12 was associated with poorer OS [44]. To concentrate on critical regions of the PPIs network, modular analysis of hub genes was implemented. We targeted highly concentrated regions as this is more productive according to drug compounding recommendations.

TF-gene interactions were identified with common DEGs. TF-genes act as regulators according to the expression of genes that may lead to the production of cancer cells. From the network, *MCM7* and *SERPINA1* showed high interaction rates with other TF-genes. The degree values of *MCM7* and *SERPINA1* in the TF-gene interaction network were 33 and 23, respectively. *MCM7* facilitates cancer procession through cyclin D1-dependent signaling and serves as a prognostic marker for patients with HCC [45]. Alpha-1 antitrypsin (AAT), a predominant plasma serine protease inhibitor encoded by serpina1, is known to promote the immune response to viral infections [46]. Among the regulators, *KLF9* and *MTA2* had significant interactions. In the TF-gene interaction network, the degree values of *KLF9* and *MTA2* were 12 and 9, respectively. Fu et al. demonstrated that mRNA and protein levels of *KLF9* were lower in hepatocellular carcinoma (HCC) tissues than in normal tissues and that upregulation of *KLF9* inhibited cell proliferation and movement [47]. Guan et al. revealed that high expression levels of *MTA2* were closely associated with advanced pathological stages and low overall survival of patients, and that *MTA2* promoted HCC proliferation and metastasis in vitro and in vivo by inhibiting the Hippo signaling pathway [48].

Regulatory biomolecules serve as underlying biomarkers in multiple complicating diseases. With this in mind, miRNAs and TF-genes were utilized to regulate the common DEGs. They were visualized and analyzed in a TF-miRNA co-regulatory network. 106 miRNAs and 104 TF-genes were identified in this study. Among the TFs with the strongest interactions, *JUN* had the high degree value of 12. *JUN* is a key regulator of inflammatory cytokine genes, such as *CCL2*, and is expressed at high levels in COVID-19 patients [49–51]. In a study by Liu et al., *JUN* was discovered to be potentially involved in the molecular mechanisms of HCC pathogenesis [52]. *SP1* had the high degree value of 10. SP1-regulated RasGRP1 transcription stimulates proliferation of HCC [53]. TF-genes are responders that perform expression regulation by binding to target genes and miRNAs and can regulate gene expression through mRNA degradation [54].

Antimycin A is considered to be effective in decreasing the activity of coronaviruses and also has the potential to treat COVID-19 [55]. It is predicted that cell surface-bound immunoglobulin (csBiP) readily binds to SARS-CoV-2. CsBiP has a peptide-binding substrate-binding domain (SBD), and acetohexamide can bind to the SBD thereby interfering with the binding of SARS-CoV-2 to csBiP [56]. Betamethasone improves symptoms of hyposmia in patients with COVID-19 [57]. Since SARS-CoV-2 is a novel virus, drug treatment has been less extensively studied thus far. For this reason, we were only able to gather a small number of samples for analysis. In the future, once more samples become available, the ongoing study will gain further validity in the context of the SARS-CoV-2 pandemic.

## Conclusions

In regards to transcriptomic analysis, no other studies on SARS-CoV-2 and HCC have been done so far. We completed an analysis of the DEGs between the two datasets, filtered the material by common gene identification and tried to identify the infection response between SARS-CoV-2 and HCC affected hepatocytes. In this study, we elucidated the intrinsic association between HCC and COVID-19 by identifying key genes that are commonly up- or down-regulated by HCC and COVID-19. In addition, this study also indicates that COVID-19 may be a risk factor for the progression of HCC. Drug targets are logically suggested because they are derived from the identification of hub genes and may serve as a positive preamble to already approved drugs. HCC and COVID-19 share common targets. We propose these targets to help scientists and clinicians better investigate the mechanisms underlying the possible complications in HCC patients with COVID-19. Given that SARS-CoV-2 is a recently evolved virus, studies of its risk factors and infection are scarce. Unique studies of SARS-CoV-2 will become increasingly significant as more data sets become available.
